# Comparative Analysis of Psychology Responding to COVID-19 Pandemic in Brics Nations

**DOI:** 10.3389/fpsyg.2021.567585

**Published:** 2021-06-03

**Authors:** Katie Moraes de Almondes, Lisiane Bizarro, Maria Cristina Oliveira Santos Miyazaki, Maria Rita Zoéga Soares, Ana Carolina Peuker, Maycoln Teodoro, João Gabriel Modesto, Aleksander N. Veraksa, Purnima Singh, Buxin Han, Tholene Sodi

**Affiliations:** ^1^Department of Psychology, Federal University of Rio Grande do Norte, Natal, Brazil; ^2^Institute of Psychology, Federal University of Rio Grande do Sul, Porto Alegre, Brazil; ^3^Psychology Course, Medical School of São José do Rio Preto, São José do Rio Preto, Brazil; ^4^Department of General Psychology and Behavior Analysis, State University of Londrina, Londrina, Brazil; ^5^Bee Touch, Porto Alegre, Brazil; ^6^Department of Psychology, Federal University of Minas Gerais, Belo Horizonte, Brazil; ^7^State University of Goiás (UEG) and University Center of Brasília (UniCEUB), Brasília, Brazil; ^8^Psychological Institute, Russian Academy of Education and Faculty of Psychology, Lomonosov Moscow State University, Moscow, Russia; ^9^Department of Humanities and Social Sciences, Indian Institute of Technology Delhi, New Delhi, India; ^10^CAS Key Lab of Mental Health, Institute of Psychology, Beijing, China; ^11^Department of Psychology, University of Limpopo, Sovenga, South Africa

**Keywords:** COVID-19, BRICS forum, psychology, telepsychology, actions, health workers

## Abstract

The BRICS Forum, an independent international organization encouraging commercial, political, and cultural cooperation between Brazil, Russia, India, China, and South Africa, was formed in 2011, and these countries have a significant influence on their regional affairs. These nations were hit by COVID-19 at different times, and all adopted home quarantine to reduce the spread of the virus. We present a comparative analysis of actions of psychology and potential outcomes during the COVID-19 pandemic in BRICS nations regarding five aspects: psychology in health policies, social roles of psychology, socioeconomic context, actions for the general population, and health professionals during stage 1 of the pandemic, and possible actions in stage 2. Various types of actions were taken by psychologists in BRICS, with different levels of coordinated cooperation with respective governmental and non-governmental organizations, multiple and parallel efforts from different scientific societies, and professional regulatory agencies. Scientific societies have had an important role in coordinating some of these efforts, especially because they congregate the psychologists from different parts of these countries, improving communication and access to key information. The aim of these actions varies from improving situational skills and competences to increase the accessibility of psychological services and provide psychoeducation and telepsychology. We will consider the social importance of these actions within these countries as a global opportunity for psychology to stage in a complex context involving human health. The way psychology in BRICS will face this challenging situation is likely to produce important regional influence, stimulate scientific contribution, and increase the accessibility of psychology.

## Introduction

BRICS is the group composed of five major emerging countries: Brazil, Russia, India, China, and South Africa, which, together, represent about 42% of the population, 30% of the territory, 23% of Global Gross Domestic Product (GDP), and 18% of the global trade. Since 2009, these countries have sought to establish fairer international governance, developed sectoral cooperation in different areas, such as science, technology and innovation, economics, finances, business, education, and security (BRICS-Brasil, 2019). The BRICS is an economic group composed of designating countries considered to be emerging, which have the economic potential to overcome the great world powers. It is not an economic block or an international institution but an international mechanism with a diplomatic character, which promotes the collective economic and political actions by these countries. Currently, sectoral cooperation, which covers more than 30 subject areas, brings important concrete benefits to the populations of the five countries.

As a major global health problem, COVID-19 is a central theme on the BRICS agenda for having an impact on the capabilities of health infrastructure and global economies, in addition to the important discussion on political management that is associated with the development of efficient actions in these countries. Psychological scientific societies of these countries are aware of these impacts, concerned with consequences for the mental health of the general population and, in particular, health professionals, with psychosocial actions aimed at different vulnerable groups (children, the elderly, people in vulnerability, and victims of violence), taking into account sociocultural differences and the particular attempt to strengthen health systems in these developing countries.

In this sense, this perspective article discusses the actions of psychology as a science and profession, represented by several scientific societies and councils, governmental and non-governmental organizations, and universities, in combating the pandemic of COVID-19, in the countries that form the alliance of the BRICS. This proposal is important, given that we are among the 25 countries that most produce science in psychology, in a list of 173 countries (https://www.scimagojr.com/countryrank.php?area=3200&year=2019). If the BRICS were a country (adding up all the scientific production would be 8,118 documents in 2019), we would be third in the scientific production of psychology, behind the USA and the UK, well-ahead of Germany. Therefore, we present an analysis of actions of psychology and potential outcomes just after the outbreak of COVID-19 in the BRICS nations regarding five aspects: psychology in health policies, social roles of psychology, socioeconomic context, actions for the general population, and health professionals during stage 1 of the pandemic, and possible actions in stage 2. Finally, we consider a comparative analysis of these actions. Before this analysis, we characterize the psychology in the BRICS countries.

### Characterization of Psychology in the Brics Countries

#### Brazil

There are 350,000 registered psychologists in Brazil today (http://www2.cfp.org.br/infografico/quantos-somos/), working in individual practice, healthcare, education, organizations, and research. Profession and formation have been regulated by Federal law No. 4,119 since 1962. Federal Council of Psychology (FCP) has been the organization responsible for professional registration and regulation since 1972. Brazilian Society of Psychology (BSP) has congregated all areas of scientific and professional psychology since 1971 (Williams and Hubner, 2014) and joined the International Union of Psychology Science (IUPsyS) in 1957.

In 2016, there were 626 courses of psychology (in public and private institutions) with 22,985 enrolled students, after the Higher Education project that intensified the expansion, democratization, interiorization, and internationalization of Higher Education (Dantas et al., 2019). Extensive supervised training during 5-year courses is mandatory in different areas of professional psychology. Therefore, universities and faculty are part of the public mental care service. Parallel to professional growth, scientific knowledge is produced in about 90 graduate courses and published internationally and by dozens of open-access national scientific journals linked to universities and scientific societies of different subfields of psychology.

Regarding mental health, SUS and Unified Social Assistance System (SUAS) have become important devices for the integration of professional psychologists in public policies since the 2000s. Both were responsible for the insertion of psychologists to the most diverse municipalities and locations in the country to a contingent, estimated in 8,000 psychologists in Reference Centers for Social Assistance (CRAS and CREAS) and 40,000 psychologists in SUS (Macedo et al., 2011). Unified Health System (SUS) serves more than 70% of the population. It works in regionalized care networks, integrating different levels of care complexity, from community Basic Health Units (UBS) to hospitals. The characteristics of the service provided vary according to the different regions of the country. In recent years, there has been an expansion of the inclusion of psychology in primary care, especially in the Family Health Strategy (ESF) and in Family Health Support Centers (NASF), where psychologists perform socio-educational activities, home visits, and clinical care. However, there is no clear role of psychology in health teams (Seidl et al., 2019), which contrasts with the traditional formation of psychologists and expectations of the users (Seidl et al., 2019).

#### Russia

In Russia, more than 200 universities are engaged in the training of psychologists. Currently, according to the reports of the respected ministries, about 60,000 psychologists work in the field of education, in the healthcare sector, more than 30,000 psychologists; in the structure of law enforcement agencies, about 40,000 psychologists; in the system of social protection, more than 25,000 psychologists; in the HR system of organizations, about 50,000 psychologists; and more than 95,000 psychologists conduct individual practice.

The largest and oldest organization that unites professional psychologists in Russia is the Russian Psychological Society (RPS), one of the oldest psychological societies in the world, which was founded in 1885. The purpose of the creation of RPS was the development of “psychology, in its compositions, applications, and history, and the spread of psychological knowledge in Russia (RPS).” Members of the society were world-famous scientists who have made substantial contribution to the formation and development of psychological science: L. S. Vygotsky, A. R. Luria, A. N. Leontiev, P. Ya. Galperin, and other leading scientists of the Russian Academy of Sciences and the Russian Academy educations, leading psychologists of the largest scientific and educational organizations. Together with the Federal Educational and Methodological Association in the Field of Higher Education, RPS organizes scientific and methodological support for the preparation of federal state educational standards of higher professional education and basic professional educational programs in the field of psychological education.

#### India

Psychology as a discipline started in 1916 when the first department of psychology was established at the University of Calcutta, India. Later, departments of psychology were established at the University of Mysore in 1924, University of Madras (1943), University of Kerala (1957), Utkal University (1958), University of Bombay (1959), Allahabad University (1961), and the University of Delhi (1964). Psychology was also introduced in the various Indian Institutes of Technology and Management setup later. During the years, various psychology associations were formed, and it is difficult to give the exact number of associations in the country. The Indian Psychological Association was established in 1925, Indian Psychoanalytic Society in 1922, and later on, Indian Association of Clinical Psychology, National Academy of Psychology (NAOP), Indian Academy of Applied Psychology, and several other associations focusing on health, school psychology, community psychology, counseling and guidance, and about 20 regional associations in the different states, besides numerous local chapters. The idea of a federation of associations has been mooted and felt important and announced in 2014, but much needs to be done. The focus of these associations has been more on academic work and awareness generation than psychological services, except for a few. The National Academy of Psychology (NAOP) is a member of the International Union of Psychological Sciences and participates regularly and actively in its programs. It is also a member of the Asia Pacific Association of Psychology and engages in collaborative endeavors, including programs in disaster management.

The Rehabilitation Council of India (RCI) regulates the professional programs in clinical and rehabilitation psychology (Brazilian Federal Government, 2020). Psychologists work in close association with professionals in allied disciplines, such as psychiatry, pediatrics, education, social work, nursing, rural development, rehabilitation, defense, and management. It may be noted that, with the realization of the complexity, the move is more toward multi- and trans-disciplinary initiatives. In India, there is no registry of psychologists in the country; hence, an accurate count is difficult. Manickam (2016) suggests that there may be more than 300 master-level programs of psychology with different specializations in the country and, perhaps, more than 100,000 psychologists based on the number of training centers and the intake at each center (p. 5). This number may have increased in the last 4 years.

India has several institutions working for the mental health and well-being of its citizens. In 2017, the country passed a progressive Mental Healthcare Act, 2017 (The Mental Health Care Act, 2017) “to provide for mental healthcare and services for persons with mental illness and to protect, promote, and fulfill the rights of such persons during delivery of mental healthcare and services and for matters connected therewith or incidental thereto” (Government of India, 2017). The act also outlines the responsibilities of other agencies such as the police concerning people with mental illness as well as attempts to tackle the stigma of mental illness and its amelioration. Many current mental health-related services happening in the country are in the backdrop of this act. NIMHANS (National Institute of Mental Health and Neuro Sciences) is the major center for mental health and neuroscience education in the country. The institute provides a large number of mental health and well-being-related services. Several other institutions both in the government sector and the private sector are providing their services for mental health care, including many mental health professionals and clinical psychologists in various medical institutions and hospitals in the country. These institutions during COVID times are providing counseling and mental health facilities for the public as well as special groups like children, migrants, elderly, etc.

#### China

Psychology as a discipline was established in China around the 1920s with several milestones achieved at that time, such as the first psychology laboratory (1917, Peking University), the first psychology department (1920, Nanjing Normal College), the Chinese Psychological Association (1921, the predecessor of Chinese Psychological Society, CPS), and the first research institute (1929), Institute of Psychology, Academia Sinica (the predecessor of Institute of Psychology, Chinese Academy of Sciences, IPCAS) (Han and Zhang, 2007). CPS is one of the earliest (seventh) national academic organizations in the world. It has 36 divisions and 12 committees for special task forces. Two other national associations of psychology are the Chinese Association of Social sychology (1982) and the Chinese Association for Mental Health (1979).

CPS joined the International Union of Psychology Science (IUPsyS) in 1980, and the International Association of Applied Psychology (IAAP) in 1984. CPS hosted the 28th International Congress of Psychology, held in Beijing on August 8–13, 2004, under the auspices of IUPsyS. CPS and CAMH, jointly in cooperation with Beijing University (Department of Psychology), held the fifth World Congress of Psychotherapy in Beijing in October 2008. In 2 years, CPS will host the 30th International Congress of Applied Psychology in Beijing, July 2022.

Psychology is taught in China at both undergraduate and postgraduate levels, in 815 universities and institutes that offer postgraduate programs, 114 have master programs in psychology (psychology, mental health education) and 30 universities have doctorial level programs in psychology in 2020 (China Education online).

In 2002, the China Ministry of Labor and Social Security launched a certification for psychological counselors, based on the National Standards on Psychological Counseling. The new certification was given to 1.2 million counselors who fulfilled the basic requirement, took the training, and passed a national examination. It enables private psychotherapy practice and psychological counseling in general public settings, such as hospitals, universities, primary and secondary schools, entrepreneurs, and communities.

#### South Africa

Regulation of the practice of medicine and allied professions in South Africa dates back to the late nineteenth century when the then Colonial Medical Council of the Cape Province was established in terms of Section 18 of the Medical and Pharmacy Act of 1891. At that time, the Cape Province was nominally under the British colonial rule, which lasted until 1910, when the Union of South Africa was formed in 1910. In 1928, the South African Medical and Dental Council (SAMDC) was appointed in terms of Act Number 13 of 1928, which was amended in 1974 (Health Professions Council of South Africa, 2020). Through a series of amendments, the SAMDC was subsequently replaced by the Health Professions Council of South Africa (HPCSA). The HPCSA consists of 12 professional boards, with the Professional Board for Psychology being one of them. The Professional Board for Psychology has the mandate to, among others, 1. Control and to exercise authority in respect of all matters affecting the education and training of persons in, and the manner of the exercise of the practices pursued in connection with, any health profession falling within the ambit of the professional board; 2. Maintain and enhance the dignity of the profession and the integrity of the persons practicing the profession (Health Professions Council of South Africa, 2020).

To practice in South Africa, a psychologist has to register with the HPCSA under any of the following categories: clinical, counseling, educational, industrial, or research. Besides, the HPCSA has provided for the registration of psychometrists and registered counselors who can be categorized as mid-level psychology professionals. Psychologists are expected to adhere to their scope of practice as outlined in the Regulations Defining the Scope of the Profession of Psychology.

One of the challenges that South Africa faces is the acute shortage of psychologists and other mental health professionals. A study by Docrat et al. (2019) has found that there were only 0.97 psychologists per 100,000 uninsured population in South Africa, between the years April 2016 and March 2017. Information obtained from the HPCSA indicated that, as of May 2, 2018, 8,773 psychologists were servicing a total population of ~57.78 million people in South Africa (Stats, 2019).

Apart from the statutory Professional Board for Psychology, there are several psychological associations operating in South Africa. The Psychological Society of South Africa (PsySSA) is the largest of these associations, with over 1,000 members (Psychological Society of South Africa, 2020). The society was formed in 1994 through an amalgamation of various psychological bodies that were in existence at that time.

## Sections Assessment of Policy/Guidelines Options and Implications

We present a comparative analysis of actions of psychology and potential outcomes during the COVID-19 in the BRICS nations regarding five aspects: psychology in health policies, social roles of psychology, socioeconomic context, actions for the general population, and health professionals during stage 1 of the pandemic, and possible actions in stage 2.

### Psychology in Health Policies

#### Brazil

In Brazil, the first case of COVID-19 was reported around February 26, and quarantine began in several cities in March. By May, some cities enacted lockdown due to a high number of deaths. Since then, 349,113 cases have been confirmed, and 22,165 deaths occurred until May 24, 2020 according to the Brazilian Ministry of Health (2020b).

The Ministry of Health issued an ordinance n 639 on March 31, 2020, named “Brazil counts on me” (Brazilian Ministry of Health, 2020c) for recruiting health professionals, including psychologists, to work in cities with a shortage of workers. The TeleSUS made available information and guidance about COVID-19, using chat, apps, a telephone, and WhatsApp, aiming at increasing quarantine of potential cases or risk groups (https://aps.saude.gov.br/ape/corona/telesus) but did not provide psychological care. Quarantine, lockdown, or other measures of social distancing were recommended at the discretion of governors and mayors but not by the Ministry of Health. In a controversial attitude, the Brazilian president not only did not recommend social distancing but also stimulated social gathering on public appearance in front of the official presidential palace, visiting shops and buying from street vendors in Brasília with national media coverage. These mixed messages concerning the severity of the pandemics were considered as a menace by the international community (The Lancet, 2020).

This was the first time that the Brazilian government has called on psychologists through “Brazil counts on me” (Brazilian Ministry of Health, 2020c). The Brazilian public health system hires psychologists to work in primary care in the Family Health Strategy (ESF) and in Family Health Support Centers (NASF), as mentioned above. However, until then, there was no work organized to serve the population at different levels of health care in biological disasters and aimed at the general population, patients, and healthworkers. Also, we did not have a tradition of online psychological care for health or training for psychologists in this field.

Brazilian psychologists have completely changed their professional activities, turning to online services. On March 26, the Federal Council of Psychology published a national resolution (Federal Council of Psychology, 2020a), reducing bureaucracy for psychologist's registration for practice mediated by information and communication technologies. For instance, during COVID-19, psychologists can make use of information and communications technology to assist people during crises or emergencies, which was forbidden before. Despite these changes, some limitations to online care were maintained so far, such as psychological assessment (Federal Council of Psychology, 2019), mainly concerning the use of some psychological tests, and, for some cases of forensic assessment, face-to-face service must be provided (Federal Council of Psychology, 2020b). The pandemic crisis has many potential sources of psychological distress (large-scale deaths, economic recession, unemployment, urban violence, lack of protective equipment, fear of contracting the virus, stigmatization). The provision of psychological services for mental health for the population, health professionals, and patients was a fundamental condition in this period (Torales et al., 2020).

Changes in regulation of telepsychology were crucial to the implementation of health policies, such as psychological care offering to the community, especially to health professionals. One of the first initiatives aimed at telepsychology to the population with mental suffering was a partnership between the Brazilian state of Rio Grande do Norte and the Federal University of Rio Grande do Norte.

Universities are providing teleservices [e.g., University of Juiz de Fora (calmanessahora.com.br); State University of Londrina (www.uelcontracoronavirus.com)]. Federal University of Rio Grande do Sul associated with the Ministry of Health organized a call center to offer psychological support to health professionals involved in COVID-19 (https://sites.google.com/hcpa.edu.br/telepsi/), using an open platform for training and research. In the same way, Oswaldo Cruz Foundation of Brasília (Fiocruz), a research institution linked to Ministry of Health, produced a series of e-books about coronavirus (https://www.fiocruzbrasilia.fiocruz.br/coronavirus/saude-mental-em-tempos-de-coronavirus/). From this material, an online course was created for several health professional categories, including psychologists. After completing this training, professionals will be allowed to use a telepsychology platform provided by Fiocruz.

COVID-19 associated with quarantine has produced changes in the psychological practices, bringing new challenges for professionals in Brazil. Researchers from the Brazilian Society of Psychology (BSP) have produced guidelines for psychologists (https://www.sbponline.org.br/enfrentamento-covid19) to guide evidence-based practice during the pandemic and a resource hub to provide links to different kinds of material related to psychological effects of COVID-19 produced by other scientific societies, researchers, and international health organizations.

#### Russia

The COVID-19 pandemic has led to significant changes in the lives of Russians. To date, according to the official information (https://xn--80aesfpebagmfblc0a.xn--p1ai/), more than 280,000 people are ill, 2,631 died. At least half of the cases detected in the capital of Russia, Moscow city, with a population of more than 12 million and over 17 million people live in the suburbs.

The self-isolation regime announced on March 30, 2020, during which, the majority of the population was compelled to stay at home, raised challenges that required a response from psychological science and practicing psychologists. Though, currently, in some regions, self-isolation regime is taken off in Moscow, and in regions with detected high numbers of infected, it was prolonged until the end of May. During the last 15 years, Russian Psychological Society became deeply integrated with the education (elaboration of the educational standards for school- and university-level education). However, a lack of legislation on psychological help in the country due to the absence of specific regulations is probably the major challenge faced before and during pandemic, when the qualification of the service of volunteer psychologists became a relevant issue.

Rise in emotional reactions associated with the virus (fear of getting sick, anxiety for oneself and relatives, etc.), changing living and working conditions (cabin fever, violation of family structure, etc.), a situation of uncertainty about both the virus, and life at the end of the pandemic regime were detected.

#### India

The first case of COVID-19 in the country was reported on January 30, 2020. This has increased to a total of 112,359 cases, 3,435 deaths in the country until May 21, 2020. So far, the majority of the cases had been reported in big cities, e.g., Mumbai, Ahemdabad, Chennai, Pune, Delhi, Jaipur. The COVID-19 outbreak was linked to people coming from outside the country initially, but, soon, it spread to many parts of the country. The Prime Minister of the country on March 22, 2020 asked the people to observe a Janta Curfew (voluntary public curfew), which found a massive support. This was a precursor to the forthcoming lockdown period. On March 24, 2020, a lockdown of the entire country was announced for 21 days, affecting the entire 1.3 billion population of India. On April 15, lockdown 2 was announced, extending the earlier lockdown until May 3, 2020, which was further extended to May 17, 2020 (lockdown 3). The Government decided to divide the country into three zones: Green Zone, Red Zone, and Orange Zone, having varying degrees of relaxations. We are now having another lockdown 4, until May 31, with more relaxations. During the lockdowns, various efforts were made by government agencies to make essential supplies available to people so that some of their hardships were taken care of.

The government launched a smartphone application called “Arogya Setu” to contain the spread of COVID-19 as well as to do contact tracing. The app intends to spread awareness of COVID-19 and to connect essential COVID-19-related health service to the people. The Ministry of Health and Family Welfare of India has created a separate section for behavioral health related to COVID-19 on its official website. The officials have actively been posting several audiovisuals, addressing the issues of social stigma related to COVID-19, stress management, depression, tobacco and alcohol consumption/addiction, practical tips on handling mental health during the lockdown, and taking care of the mental health of children as well as elderly. The Ministry early on undertook the role to create greater awareness of important issues, such as how to handle social isolation and has provided some practical tips to handle emotional problems, addressed “psychosocial issues among migrants during COVID-19,” among others. These efforts came as early as the last week of March, the week when the lockdown was announced for the first time in India. More and more resource material is actively being added to the repertoire. A toll-free helpline number has also been issued, specifically to deal with psychosocial difficulties/issues (Ministry of Health Family Welfare Government of India, 2020).

A relatively young Ministry of AYUSH established in 2014 has also been playing an active role in the dissemination of information such as how to boost immunity and has provided simple ayurvedic procedures for cough and sore throat (Ministry of AYUSH, 2020). Ayurveda has for long played a central part in many people's beliefs and attitudes toward health issues in India. Positive informative messages from an official government ministry at a national level can help in boosting people's sense of security in these uncertain times.

The National Disaster Management Authority of Government of India on their home page created a subsection “COVID-19: Positive Stories” wherein various uplifting stories of generosity, positive developments in handling the COVID-19 virus, among others, are continuously published (National Disaster Management Authority, 2020a). Among the plethora of advisories from various ministries and departments of Government, the need to create social support and take care of the mental health of frontline workers was not forgotten (National Disaster Management Authority, 2020b).

However, government measures in dealing with mental health issues beyond providing resource materials and advisories are minimal. The fact that such issues have been acknowledged by the government is a positive step but still insufficient. The biggest concern in India is the well-being of the large section of the population, who are at the periphery. These include women, children, minority groups, other deprived groups based on caste and class as well as the physical and mentally challenged persons. Psychological associations face the biggest challenge to provide mental health services to this section. Several NGOs have also been very active. During COVID-19 times, these vulnerable groups have been worst impacted, challenging the unorganized psychological services in the country in many ways.

Using the Ministry of Health and Family Welfare's data source, NDMA created a list of COVID-19 warriors that total to 13,968,832. Among these are psychologists subsumed under allied and healthcare professionals at a paltry number of 532 and psychosocial care personnel that include M.Sc. psychology students and master of social welfare students with a total number of 1,163,46 (National Disaster Management Authority, 2020c). These figures do indicate that there is scope to further accept and strengthen the role of psychology in mental health services during these ties.

#### China

The CPS developed its Registry System for the Chinese clinical psychologists (RSCP) and set up criteria for qualification, continual education and training, and ethical standards (published in 2007, revised 2018) (Clinical Registration System of the Chinese Psychological Society, 2018). Soon after the COVID-19 breakout in early January of 2020, Chinese psychologists actively provided three levels of psychological assistance. At the first level, suggestions were provided to government agencies to take necessary actions to prevent public panic and promote psychological relief from the beginning of the blockage. The psychological intervention has been integrated in the national emergency administration from the early stage. On January 26, China CDC issued the guideline of emergency psychological crisis intervention during the coronavirus outbreak. On 5th March, National Health Commission and the Ministry of Civil Affairs jointly issued a guideline to call for psychological assistance and social services for patients of the COVID-19, quarantined persons and frontline workers in epidemic prevention and control (Zhengkui et al., 2020).

Health administration and civil affairs departments in severely affected regions should organize professional teams to provide online and offline psychological assistance for frontline medical workers and disease-control personnel, as well as those who stick to their posts, including traffic police, logistics, and community workers, according to the guideline. On March 18, the State Council interagency task force issued “psychological counseling for those affected by COVID-19 (Jia et al., 2021), which called for sustained psychological counseling services, especially for COVID-19 patients, their families, families of fallen patients, vulnerable groups, health workers and those fighting the virus in the front line, including police officers and community workers. The psychological services (including lectures for general populations, individual counseling, and online self-helping programs-APPs) are mainly provided remotely via the internet or mobile phones (Li et al., 2021). Two weeks after the quarantine of Wuhan, the epidemic center of the COVID-19 breakout, on February 7, the National Health Commission released the guideline for psychological intervention hotlines during COVID-19, guiding the hotlines' setup, counselor training, and supervision. The guideline also addressed the importance of ethics in the distance counseling. Psychologists (especially those in the CPS Division of Clinical Psychology) have been working closely with governmental officials and psychiatrists and social workers at different levels. It is necessary to incorporate psychological support into the planning and construction of social governance and social psychological service system. Mental health of common civilians and the needs of a stable and harmonious society continue to drive the development of psychology in China.

From the nature of different subjects and a country's requirements of social psychological service system, group-oriented and individual-oriented social psychology are inevitably apart in many aspects, as service recipients, service concepts and ideas, service implementation, etc. It is recommended to build the social psychological service and social governance system based on social psychology, instead of only from the perspective of individual psychological health.

Traditional Chinese culture emphasizes more on group rather than individual. It is easier for group-oriented social psychology to study and design the construction of the social governance and social psychological service system from a macro level. Praise to the Traditional Chinese Medicine, together with the western medicine, the COVID-19 pandemic had been actually contained in Wuhan (and all around China) in late March 2020. However, the home quarantine policy had been strictly, whenever required. Psychological supports have been provided systematically throughout China at the community level, with focuses on arch hospitals and schools/universities, mainly via online services.

All the above highlight and reinforce the importance of group-oriented social psychology in the construction of social governance and social psychology service system.

#### South Africa

As of May 23, 2020, the total number of people infected with COVID-19 was 21,343. This survey is updated regularly on the South African government website that was launched specifically to communicate information about the pandemic [see SA Coronavirus (n.d.)].

On March 15, 2020, the South African Minister of Cooperative Government and Traditional Affairs declared a National State of Disaster in terms of the Disaster Management Act Number 57 of 2002. Through the National Disaster Management Center, which is provided for in the Act, the government is required to “promote an integrated and coordinated system of disaster management, with special emphasis on prevention and mitigation by national, provincial, and municipal organs of state, statutory functionaries, (and) other role players involved in disaster management and communities” (Department of Cooperative Governance and Traditional Affairs, 2002).

Informed by this Act, the South African national government has welcomed the involvement of individuals and professional groups that have offered to assist the government in its response to COVID-19. Consequently, psychologists in South Africa have individually and collectively come forward and are now involved in efforts spearheaded by the government to contain the pandemic. For instance, the Psychological Society of South Africa is one of the organizations that have formed what is known as “HealthCare Workers Care Network (HWCN),” an organization made up of psychologists, psychiatrists, anesthesiologists, medical doctors, and other health professionals. This multi-stakeholder initiative aims to support health care workers during the COVID-19 pandemic and beyond (Psychological Society of South Africa, 2020). Currently, this organization provides training, webinars, and psychoeducational information for healthcare workers. It also uses volunteers to offer individual and group support to healthcare workers. All mental health initiatives that are influenced by the Disaster Management Act are likely to have important psychosocial benefits for vulnerable groups, such as homeless people, the elderly, children, and those exposed to violence, drugs, and substance abuse.

At the beginning of May 2020, the country transitioned into Level 4 of the five-stage risk-adjusted response, with Level 3 due to start on June 1, 2020. As shown in Table 1, Level 4 provides for extreme precautions to limit community transmission and outbreaks, while allowing some activity to resume. Some of the activities that were allowed during this level were limited wholesale, retail, manufacturing, and agricultural and health activities. While less severe, Level 3 will still impose restrictions on work and social activities to address the high risk of transmission. Some high-density settlements will be declared hotspots during Level 3. A hotspot is defined as “… an area that has more than five infected people per 100,000 people or where the new infections are increasing at a fast pace” (President Ramaphosa speech, May 24, 2020).

**Table 1:** Summary of alert levels in South Africa [adapted from SA Coronavirus (n.d.)].

**Alert level**	**Level 5**	**Level 4**	**Level 3**	**Level 2**	**Level 1**
Objectives	Drastic measures to contain the spread of the virus and save lives	Extreme precautions to limit community transmission and outbreaks, while allowing some activity to resume	Restrictions on many activities, including at workplaces and socially, to address a high risk of transmission	Physical distancing and restrictions on leisure and social activities to prevent a resurgence of the virus	Most normal activity can resume, with precautions and guidelines followed at all times. Population prepared for an increase in alert levels if necessary

Due to the restrictions imposed on individuals during the pandemic, the Health Professions Council of South Africa (HPCSA) had to revise its guidelines on the provision of online health services. In its original General Ethical Guidelines for Good Practice in Telemedicine, the HPCSA discouraged health practitioners from routinely servicing their patients virtually (Health Professions Council of South Africa, 2020). In a recent notice issued on March 26, 2020, in response to COVID-19, the HPCSA pronounced that “telehealth is only permissible in circumstances where there is an already established practitioner-patient relationship, except where telepsychology and/or telepsychiatry is involved; in which case, telehealth is permissible even without an established practitioner-patient relationship” (https://www.hpcsa.co.za/Uploads/Professional_Practice/FAQ_Professional_Practice.pdf). This new development provides an opportunity for psychologists to explore new avenues for providing mental health services for their clients, now and possibly, in the future. As a result of this reform, many telehealth platforms are now inviting health professionals to attend webinars that offer some form of training on how to provide telehealth services, including a teletherapy and counseling (for example, see www.ezmed.solutions and www.cgm.com/za). The medical insurance companies have also come out in support of telehealth services by assuring psychologists and other health professionals that they will reimburse the service providers for this alternative service [for example, see Discovery Health (n.d.)].

It is important to register that 26 years after the demise of a colonial government that guaranteed more privileges for a minority community of European descent when compared with indigenous African communities, the majority of South Africans continue to have little or no access to mental health services. One of the key focus areas of the Psychological Society of South Africa before COVID-19 has been to actively strive for social justice by opposing policies and practices that deny individuals or groups access to mental health services.

### Social Roles of Psychology

#### Brazil

The COVID-19 pandemic has exacerbated social inequalities (Dorn et al., 2020) in different countries, and Brazil is no exception. Considering that one-third of households do not have adequate sewage collection, 15.1% do not have running water (Brazilian Institute of Geography and Statistics, 2018) and the high number of homeless people and prisoners who cannot be in social isolation, basic preventive care is not available to everyone. Besides hampering basic care, inequality *per se* tends to intensify during the pandemic. Prejudice (Bavel et al., 2020) and domestic violence (Marques et al., 2020) are expected, among other implications. In this sense, psychologists should be able to offer psychosocial support to those facing inequalities during this period. CRP and its regional councils advised psychologists on the importance of psychosocial support for different vulnerable groups, such as homeless people (Regional Council of Psychology-São Paulo, 2020) and prisoners (Regional Council of Psychology-Bahia, 2020; Regional Council of Psychology-São Paulo, 2020). Moreover, scientific societies, universities, and different groups of psychologists have mobilized to deal with other social problems, such as prejudice, domestic violence, and fake news.

##### Prejudice

Prejudice is a hostile attitude toward groups or individuals for being part of certain groups (Allport, 1954). During the pandemic, hostility toward Chinese has grown, even promoted by some Brazilian politicians (Globo, 2020). There are also reports of health professionals being stigmatized (Markman, 2020). Seeking to reduce the occurrence of prejudice and discrimination, the Brazilian Psychological Society prepared a document with guidelines for facing stigma and prejudice during the pandemic (Peuker and Modesto, 2020).

##### Domestic Violence

Domestic violence has been one of the consequences of social distancing (Chandan et al., 2020). In Brazil, it is estimated that, in March (when social distancing measures were announced in the country), the increase in domestic violence was 17% (Marques et al., 2020). Seeking to reduce the problem, scientific societies and groups of researchers have sought to inform psychologists and the general population about the different types of domestic violence (Curia et al., 2020), conflict mediation strategies in the family (Soares and Modesto, 2020), as well as channels to Brazilian institutions that offer psychosocial support (Mello and Modesto, 2020).

##### Fake News

Besides social inequality, the spread of fake news in social media became another problem for public health in Brazil (Thomas et al., 2018; Waszak et al., 2018). Also, the Brazilian president took a stand against social isolation and minimized its consequences, which has been a problem for the country, according to an editorial in The Lancet (2020). Brazilian researchers analyzed the influence of fake news on prevention behaviors during the pandemic and found out that belief in fake news was associated with less social distancing (Modesto et al., submitted)^1^, although political polarization has a more robust effect than fake news (Modesto et al., 2020). Many scientific societies in Brazil have shared WHO, PAHO recommendations, as well as producing accurate material that aims to confront fake news.

#### Russia

While organizing psychological assistance in the usual ways (in person) is not possible, the possibilities of online counseling are widely used. The Russian Psychological Society, the Russian Academy of Education, Faculty of Psychology, Lomonosov Moscow State University organized several hotlines for psychological assistance [including a united 24/7 hotline on the website of COVID-19, organized by the Russian Government (https://xn--80aesfpebagmfblc0a.xn--p1ai/)]. In total, more than 1,000 psychologists participate in the work of hotlines of federal and regional significance.

#### India

The role of psychology is immense in these circumstances. Several psychologists, as well as NGOs involved in elderly care, homeless people, and vulnerable sections of the population, are actively participating in providing relief and mental health services. Psychology associations are playing an active role as well as psychologists and healthcare professionals associated with medical institutions. For instance, a relatively new Association of Health Psychologists (AHP) took an active initiative in extending its services during the time of COVID-19. It was the first professional organization to launch online counseling in the first week of April 2020 for anyone directly or indirectly affected by COVID-19 or the lockdown-related psychological issues. The first initiative was in Hyderabad under the banner of “SERV,” an acronym for “Social Emotional Rehabilitation of Virus Victims.” This was launched in collaboration with UNICEF, Action Aid, Dr. Reddy Foundation-School Improvement Programme, APTS Social Service Forum, and Asha Hospital. Counselors with the highest academic background and years of experience are available on a helpline 24 × 7. Encouraged by the response, the AHP started similar helplines in collaboration with Sri Padmavathy Mahila University Thirupathy, Andhra Pradesh, Orissa Central University, Koraput, Odisha, Calcutta University, West Bengal and Bangalore, Karnataka in collaboration with UNICEF. The counselors attended thousands of calls from different parts of the country as well as a few calls from Indians residing in other countries. In initial months, the calls related to general anxiety of the “fear of unknown”; insomnia; interpersonal conflicts due to home confinement leading to a situation called “home crowding”; the anxiety of frontline health care workers; the psychological distress of people in quarantine centers; panic attacks of people of all ages, gender, and geographical location; anxiety of students due to uncertainty of examination schedule; financial problems; non-availability/accessibility of regular medicines due to lockdown; precipitation of pre-existing mental health problems; issues related to migrant labor; and also calls from victims of domestic violence, to name a few. Apart from COVID-19/Lockdown-related problems, the helpline has been receiving calls related to other psychosocial problems. Because of this, the helplines are continuing through, and the number of COVID-19-related calls has been gradually reducing.

Several initiatives were taken by other associations as well (which will be discussed in a later section); however, looking at the population of the country, the problems are immense and can be tackled only with the combined efforts of all. More and more psychologists should be involved by the government in these times as in when there is a second wave of COVID-19 later in the year, mental health concerns are going to become pivotal. Many people have lost their jobs, and as time passes and they do not get economic relief, mental health concerns are going to become acute. It is not to say that mental health concerns are not important now. The concerns because of lockdown are related to fear, anxiety, social isolation, lack of human stimulation, working from home, and making the fine balance between home and work. Once the lockdown is lifted, as no society can be in a state of complete lockdown, the fear is related to a surge in cases of corona infections, disease, death; all of which may lead to serious mental health concerns. Psychology associations should make the government start focusing more on mental health issues of the most vulnerable sections of the society.

##### Fake News

News items and images not related to COVID-19 times were shared and created fear and panic among citizens. The government is tackling the problem of fake news by requesting social media platforms to remove and not promote messages that will weaken the government's efforts during these times. The media is continuously being requested to play a very responsible role and help in countering fake news. Some news channels are doing a commendable job of alerting people to fake news. In these times, responsible sharing of news has to be followed, but, as is the current situation, fake news is rampant. Irresponsible forwarding of messages on social media should be avoided. The Government of India has recently issued a directive, asking social media companies to voluntarily curb fake news and misinformation related to the coronavirus on their platforms. This is considered as an important step to prevent the community transmission of the disease. A lot of psychology research shows that, in times with a lot of uncertainty, rumors and propaganda of various kinds have easy acceptability among the masses. Stern and prompt actions are needed; otherwise, it will play havoc with people's life and health. Together with the government, it is also important that we, citizens, not only exercise caution but debunk such messages.

### Socioeconomic Context

#### Brazil

About 13 million Brazilians live in favelas. Hygiene recommendations are near impossible to follow in these environments because of the high demographic density and lack of treated water. Communities in favelas have organized themselves to implement measures on their own (The Lancet, 2020). The prevalence of deaths and contamination by Sars-Cov-2, inconsistent government measures of social isolation, quarantine and lockdown, and their long-term impact will widen such existing socioeconomic disparities. Therefore, the impact of COVID-19 on mental health is also greater in economically disadvantaged populations (Frasquilho et al., 2016; Mental Health Foundation, 2020; Nações Unidas Brasil, 2020). The universal income program Bolsa Família was established in 2003, and today, it serves 13.1 million families (40.8 million people), who receive an average benefit of R$ 188.86 per month. In the crisis caused by COVID-19, an emergency aid (R$600, equivalent to U$100, for 3 months) was made available to informal workers, individual micro-entrepreneurs, self-employed, and the unemployed; 60 million citizens requested it (https://www.gov.br/pt-br/servicos/solicitar-auxilio-emergencial-de-r-600-covid-19).

Brazil has a large informal employment sector, with many sources of income no longer an option (The Lancet, 2020). Considering the economic impact of the pandemic in Brazil, a survey carried out by the National Confederation of Industry (CNI) in May 2020, with 2,005 people over the age of 16, in 27 states, found out that Brazilians lost their purchasing power; 23% of respondents lost their income, and 17% had losses in their monthly income, which represent almost 40%. The fear of losing a job was reported by almost 80% of the respondents, and 9 out of 10 people rated the impact of COVID-19 on the Brazilian economy as significant. According to the data, 40% of the respondents reported having overdue accounts in the last 40 days (Agência Brasil, 2020).

With around 55% of the entire global population without access to social protection, these losses will harm many sectors, such as education, human rights, and in the most critical cases, basic food security and nutrition. Hospitals with limited resources (i.e., lack of personal protective equipment for health professionals), deficient health systems are expected to collapse. This could worsen with a peak in the number of cases since up to 75% of the population in the least developed countries do not have access to hygienic supplies. Other social conditions, such as precarious urban planning and overpopulation, deficient waste management services, and even traffic jams that prevent access to health care, can contribute to drastic growth in the number of cases of COVID-19 (Nações Unidas Brasil, 2020). In this context, it is crucial to take into account the broad socioeconomic consequences of pandemic crisis, which will have serious impacts on mental health by increased rates of unemployment, financial insecurity, and poverty (Barr et al., 2015; Holmes et al., 2020).

Many private companies have decided to help various segments of society, with hygiene items, food, clothing, and water donations, supported by the tax incentive laws. One example of organized action of the private sector is Todos pela Saúde (https://www.todospelasaude.org).

COVID-19 initially reached large urban centers in Brazil, concentrated in the industrialized state of São Paulo. However, it has advanced rapidly across the country, hitting badly the states of Amazonas and Pará and extending to indigenous territories in the Amazon. Amazonas is the Brazilian state with the largest number of indigenous people. According to the IBGE, the state has 183,514 indigenous people; of which, 70% live in villages. Many communities are lacking basic hygiene items (soap, treated water) to reduce the risk of contagion. People also live in villages and share utensils, which increases the chance of contamination. Indigenous communities also live in areas where there is limited access to healthcare, particularly intensive care beds (BBC News, 2020). Federal government has been ignoring or even encouraging illegal mining and logging in the Amazon rainforest, and, now, these loggers and miners risk bringing COVID-19 to remote populations (The Lancet, 2020). As contingent action, the Federal Government sent financial aid to the State of Amazonas. By the end of May 2020 will be inauguration of the first health area dedicated to the care of indigenous patients with COVID-19 in the State of Amazonas. It will have 53 beds, 33 clinical beds, 10 Intensive Care Units (ICU), and five Intermediate Care Units (ICU). There will also a space for spiritualization destined to shamans—spiritual leaders of indigenous peoples (Ministério da Saúde do Brasil, 2020).

#### India

One of the major social problems that have been highlighted during COVID-19 times is the sharp divide between the haves and the have-nots, the rural and the urban, and the privileged and the vulnerable sections of the society. This divide was already there but got highlighted during this time. Visuals on television of thousands of migrants fleeing from major cities to their villages are very disturbing. With factories and workplaces shut down, many migrant workers were left with no food, money, and shelter. There was thus a mass exodus from big cities. Because of the lockdown, this was a major issue. The government has been arranging relief camps for these people, announced an economic package for them and requested companies/employers to pay the workers their salary during the lockdown period. Unemployment has risen and lots of migrant workers had no choice but to return to their homes. There are some apprehensions associated with this exodus to the rural areas. As a large number of migrant laborers are returning from big cities which are the hot spots for COVID-19, the fear of the pandemic in rural areas is frightening as health infrastructure is insufficient in these areas.The lived realities of the past few months were tumultuous and catastrophic for most migrant workers, especially women, and, unfortunately, mental health, and psychological services available to them are at the bare minimum, except those provided by some NGOs. What is of utmost importance in the Indian scenario is to compile data related to psychological services and psychology professionals, as, at present, in the absence of such documentation, it becomes difficult to comment on the role of the psychological associations during this pandemic.

#### South Africa

The South African government imposed a national lockdown that lasted for 6 weeks, starting on March 26, 2020. In his address to the nation, President Cyril Ramaphosa pointed out that the national lockdown is one of the five alert levels of the South African government's risk strategy to respond to the spread of COVID-19. As reflected in Table 1, alert Level 5 of the national lockdown was the most stringent measure that entailed the closure of schools, businesses, and recreational facilities, except essential services. This alert level had a significant negative impact on the health, social, and economic lives of individuals and communities. For instance, a national survey by the Human Sciences Research Council (HSRC) found out that between 45 and 63% of those surveyed reported that the lockdown would make it difficult to pay bills, debts, earn income, feed their families, and keep their jobs (Human Sciences Research Council, 2020). The restrictions on people's movements also meant that some patients who needed to receive medical and psychosocial services could not access these, as public transport was not readily available.

The national government put in place some socioeconomic measures that were aimed at individuals, families, and businesses to mitigate against negative impact of COVID-19. At individual and family levels, the interventions introduced included payment of a social relief grant, a distress grant to the unemployed, and an increase in the social grant amount paid to the indigent, among others. For businesses, the government introduced tax relief, economic stimulus measures, and employment-related measures, such as the employment tax incentive that encourages employers to hire young unemployed job seekers. While these measures have assisted greatly in minimizing the negative impact of the pandemic, there have been several challenges associated with these government initiatives. These include a lack of capacity on the part of national and local governments to roll out so many socioeconomic measures in a short space of time.

### Actions of Psychology for the General Population and Health Professionals During Stage 1 of the Pandemic

#### Brazil

The Conselho Federal de Psicologia, the regulatory agency or professional psychology, registered psychologists for online practice and provided basic professional guidelines for work during COVID-19 (e.g., to provide services in ventilated places, allowing a distance of 1 to 2 m between people). If a professional chooses to provide online psychological services, he or she must follow previous guidelines (CFP Resolution No. 11/2018) and register to do so in the respective Regional Psychology Council.

The Brazilian Society of Psychology (BPS) constituted a Working Group of professional researchers who sought to base their actions on developing strategies for training and supporting this professional category in coping with the situation of COVID-19. The work was developed using scientific and technical criteria, using evidence to support their decisions and guidelines. The first step was to publish fascicles, available free online at the SBP homepage (https://www.sbponline.org.br). Moreover, links with relevant information about the pandemic from reliable sources were also made available by SBP.

So far, 10 topics have been developed with updated information to contribute to the professional practice of psychology. The texts are brief and objective and seek to help understand the context, identify the concepts involved, learn about alternatives, and monitor interventions. The first edition addresses the technical guidelines for the work of psychologists in the context of the COVID-19 crisis and contextualizes the pandemic and provides guidelines for psychologists to work with health professionals. The second topic entitled “Stress in Health Professionals Who Care for Patients with COVID-19,” defines stress and presents clinical examples of professionals who work with patients with COVID-19. It identifies its possible manifestations, individual and institutional management, as well as the monitoring of the interventions performed. In the third topic, the three Ds are addressed: despair, helplessness, and hopelessness in health professionals. Key D concepts, individual reactions, and examples of verbalizations of fear, despair, helplessness, hopelessness, and suicidal ideation are defined. They present factors that determine the way people face a crisis (previous experience of crisis, support system, and history of mental disorders) and the first psychological aids: welcoming and active listening, psychoeducation, helplessness approach, focuses on despair, coping fear, focus on hope, problem-solving, assessment of ideation, and suicide risk. The fourth topic deals with the stigmatization of health professionals. It contextualizes the problem, defines stigma (internal and external), and provides management strategies, such as access to information, awareness, and ethical posture. In the fifth topic, recommendations are presented for the on-line and online professional practice of psychology in the face of the COVID-19 pandemic. It addresses the alternatives on how to offer the first psychological help to health professionals working in hospitals. It guides through how to proceed with online, voluntary, in-person, and hospital care. The sixth topic deals with mourning and reviews the concept, demonstrating the physical and emotional reactions experienced in this process and the alternatives for the psychologists to address this theme. In the seventh topic, issues related to the management of conflicts in the family are presented. It discusses how isolation predisposes to the appearance of conflicts and presents strategies for handling the situation, with steps for resolving conflicts. The eighth topic discusses the management of sleep disorders in the context of coping with COVID-19. It demonstrates how the pandemic can predispose to sleep disorders and identifies management strategies. The ninth topic presents the theme of psychological support for parents of children from 0 to 11 years old during the COVID-19 pandemic. It seeks to provide support for the psychologists to work with parents and provides behavioral management strategies during isolation. The 10th topic addresses issues related to violence against women. The different forms, phases, and consequences of this type of violence are defined. Alternative actions are presented, as well as techniques and devices that are usually used in the situation.

The TelePSI Project (https://sites.google.com/hcpa.edu.br/telepsi/) is an initiative of the Ministry of Health of the Brazilian Government, in partnership with the Hospital de Clínicas de Porto Alegre, which received the support of several academic institutions and societies, including the Brazilian Society for Psychology. In TelePSI, contracted psychologists and psychiatrists offer psychological and psychiatric consultations to manage stress, anxiety, depression, and irritability in health professionals and students, teachers of all levels of education, and professionals of essential services. They receive specific online training and supervision, and data are collected to compare the effectiveness of different interventions are clinical and scientific supervision group for evaluating different types of psychotherapy for emotional problems during the pandemic, and disseminating psychoeducational materials. Oswaldo Cruz Foundation of Brasília (Fiocruz) planned a similar teleservice provided by trained volunteer psychologists.

#### Russia

##### Psychoeducation

Psychologists have been actively involved in informing the public about how to organize their lives in conditions of self-isolation with children, the elderly, alone, etc. RPS has elaborated recommendations for various groups of the population on organizing the activities during the period of isolation (http://xn--n1abc.xn--p1ai/news/themes/8462/), as well as their promotion through Federal and regional media regularly. During 2 months, representatives of the Russian Psychological Society made more than 150 interviews to the media and more than 30 interviews on Federal and Regional TV channels in the frame of talk shows, expert talks, etc., making recommendations for the public concerning the organization of the daily routine, coping with anxiety and stress, organizing activities for children, and exposing relevant fake news.

Starting from March 16, 2020 students of all higher educational institutions in Russia were urgently transferred to distance learning, which created a lot of organizational and psychological difficulties and became a source of stress for all the participants of the educational process. This shift toward distant learning was successful due to such well-established platforms as distant.msu.ru, webinar.ru, etc.

Due to anonymity, online education at the university led to cases of cyberbullying, cybertrolling, spamming, which became the reason for the development by the RPS of recommendations addressed to university teachers on how to behave properly in such situations to protect themselves and students from cyber interference.

A separate task for psychologists in the field of education was maintaining the motivation of students who were torn out of the usual educational context. The recommendations developed by the RPS allowed teachers to support the students' engagement in distance learning situations. At the same time, the psychological services of the universities monitor the psychological state of teachers who find themselves in a situation of increased stress.

No less challenging was the situation and the organization of the distant educational process at schools and preschools. The transition to distance learning became possible, thanks to the previously introduced Russian Electronic School (https://resh.edu.ru/), Moscow Electronic School (http://mes.mosedu.ru/present-en/), which is an official all-Russian resource for schooling as well as other resources, such as Yandex.Uchebniki (https://education.yandex.ru/home/), ≪Advance≫, ≪iSpring≫, ≪InternetUrok.ru≫, ≪Unicraft≫, etc. The actual collision of the norms of family life and the norms of the school in the situation of the distant organization of the educational process led to conflicts between teachers and parents, and increased stress. RPS has developed relevant recommendations for both teachers and parents to optimize the organization of the educational process and normalize relations between family and school.

##### Work With Medical Staff

In April 2020, psychologists faced the challenge of implementing psychological assistance for medical personnel experiencing a high workload and stress. Representatives of the Russian Psychological Society provided methodological assistance in the systemic psychological support of management, medical personnel, patients, and their relatives in medical institutions for patients with COVID-19. Telepsychology was organized both for medical personnel and for patients and their relatives. However, in special cases, offline work was organized.

Leading specialists of the Faculty of Psychology of the Lomonosov Moscow State University, the Russian Academy of Education, and the Russian Psychological Society, together with the Federal Medical Biological Agency of Russia, and, personally, its head, Veronika Skvortsova, had developed “psychological thermometers” for adult patients and patient-children and medical staff who provide medical care for patients with COVID-19 (http://fmbaros.ru/psikhologicheskaya-podderzhka/detail/?ELEMENT_ID=38750).

With the help of such “psychological thermometers,” one can independently measure emotional state in an online format and get instant feedback on self-help measures, the required support from colleagues, and, also, the need to seek professional psychological help in the respected psychological service of the hospital or medical center where the person is working or receives treatment.

#### India

As already mentioned earlier, the major psychology associations are making significant contributions furthering the role of psychology as a science and profession in the country. We have three major associations: National Academy of Psychology, India (NAOP, India), which represents India in the IUPsyS; the Indian Association of Clinical Psychology (ICAP); and Indian Academy of Applied Psychology (IAAP). As discussed by Manickam (2016), together these three associations may have a membership of over 4,000 psychologists, which, certainly, do not match the vast requirements of the country. The Indian Association of Clinical Psychology has been making pioneer contributions during COVID-19 times. Indian Association of Clinical Psychology (IACP) has taken an initiative to form a “COVID-19 Psychological Support Group.” This initiative is providing free telephonic/online counseling for people in emotional distress. As a part of this program, they are training volunteers so that they can help in providing psychosocial support for the callers. The association is also providing psychoeducational tips for parents/caregivers of person with disabilities. Modules on how to handle emotional and behavioral problems, behavioral management, and coping with COVID-19-related stress are also being provided for the general public. The association is also providing various kinds of psychological support for migrant workers who are facing difficult times as well as frontline workers (doctors, nurses, police personnel, etc.), who are working hard under difficult circumstances to provide health care. The Indian Academy of Applied Psychology (IAAP) has been providing online support, educating people with authentic information about COVID-19, extending help, counseling, and giving appropriate referral services to manage stress. In addition, the association is organizing special events like essay competitions and other activities related to the psychological impact of COVID-19 to increase awareness about the disease. The National Academy of Psychology (NAOP, India) has been extending support by disseminating information related to prevention and protection through webinars on issues related to pandemic and vulnerability during these times. Online support is also being provided through counseling. Besides these major associations of the country, a large number of NGOs and other associations are also contributing significantly in terms of providing mental health services, training the counselors to provide help for those who are in desperate need of assistance.

India's experiences in the pandemic of COVID-19 have shown that a better public health system with special programs based on various sections of the society is needed (Kumar et al., 2020). Though it started as a disease in urban areas, the fear of the disease spreading to rural areas is a big one. In a country like India, where 65–68% of the population live in rural areas and these areas already have the highest overall burden of disease and accompanied lack of health facilities, it is a major risk factor. As Kumar et al. (2020) argue there is the need to take immediate steps to control the spread and its aftereffects and to use this opportunity to strengthen and improve its primary health care system in rural India. The health care system is not adequate or prepared to contain COVID-19 transmission in the rural areas because of the shortage of doctors, hospital beds, and equipment, especially in densely populated states (Mitra, 2020).

As Nayar et al. (2020) posit, it is important to understand the forms of emotional disturbances among the different age groups as well as different sections of the society. A new form of “othering” is seen in society. Who is the other is being redefined. The same colleague, domestic help, and neighbor are now seen as the other due to the suspicion that they may be potential carriers of infection. There is a kind of dichotomized world view that is prevalent (Nayar et al., 2020), which has altered the fabric of the society where everyone is fearful of getting infected, despite the “not me” syndrome. Additionally, scores of people have lost their sources of income. For most people, priorities in life are changing, putting an additional burden of stress.

#### China

The Clinical Psychology Registration Work Committee of the Chinese Psychological Society (hereafter referred to as the “Registration System”), as a self-regulated professional organization of clinical and counseling psychology, devoted itself to the psychological aid after the outbreak of the COVID-19 pandemic and explored effective approaches and methods for psychological aid!

1. Speak up as professionals. The Registration Work Committee started to work on January 23 and issued a written proposal to all the psychological professionals across the nation on January 26, and advocated that all the psychological aid should be integrated in the national framework in fighting the COVID-19, and underlined the need of multi-department cooperation. It reaffirmed the professional ethics and strongly emphasized that all the psychological aid should be conducted scientific orderly and conform to professional standards.

2. Set up an organizational structure. The Registration Work Committee set up the organizational structure on January 25 and established several working groups with different functions to ensure orderly development, including the supervision group, the popular science dissemination, the information resource acquisition, and so on.

3. Give full play to the superiority of the Registration System members. A group of 161 registered clinical psychology supervisors, who were willing to supervise, was released to the public on the WeChat Official Account, providing free supervisory resources for counselors, and psychotherapists.

4. Enhance the competencies in psychological aid. The sudden outbreak of the COVID-19 emerged in a fast spread and affected widely, and as a result, most psychological aids were conducted through hotlines, including the popularization of the medical knowledge of the COVID-19. The Registration Working Committee decided to work in a pyramid model due to the specialties of the COVID-19, referring to training registered psychology supervisors and providing guidelines and approaches for them to supervise counselors and therapists who work in the front line. From January 28 to May 20, a total of 32 training sessions and supervision have been arranged for registered supervisors with a total of more than 1,600 participants. Nearly 10 million people received training and supervision focused on hotlines, psychological stress, and crisis intervention provided by the trained supervisors.

5. Timely release professional guidance documents as a leading professional organization. Guidelines for Psychological Aid Hotline under the Contagion Fight, Guidelines for Online Psychological Aid under the Contagion Fight, Ethics for Psychological Hotline (First Draft), and Ethics for Online Counseling (First Draft) were released continuously from January 31, leading professionals to conduct within competencies and comply with the ethics.

6. Cooperate with governmental officials and peer colleagues from other professionals (social workers, psychiatrists, etc.). To cooperate with the National Health Commission, the competent government department for psychological aid during the COVID-19 pandemic. The Registration Work Committee put forward targeted psychological aid policies for the country and the specific population affected by the epidemic, for e.g., timely proposing the overall plan of psychological aid construction for the Arch hospitals built in Wuhan. Besides, it joins hands with other academic organizations, such as the Chinese Association for Mental Health, China Association of Social Psychology, in fighting against the COVID-19 pandemic to share resources and joint proposals.

7. Promote the popular science on the major media. Make knowledge dissemination of audio or videos programs on how to promote mental health in the pandemic period and provide the public with knowledge and methods on mental health to adjust the mentality and be positive and optimistic.

#### South Africa

At the beginning of May 2020, the country transitioned into Level 4 of the five-stage risk-adjusted response, with Level 3 due to start on June 1, 2020. As shown in Table 1, Level 4 provides for extreme precautions to limit community transmission and outbreaks, while allowing some activity to resume. Some of the activities that were allowed during this level were limited wholesale, retail, manufacturing, and agricultural and health activities. While less severe, Level 3 will still impose restrictions on work and social activities to address the high risk of transmission. Some high-density settlements will be declared hotspots during Level 3. A hotspot is defined as“*… an area that has more than 5 infected people per 100,000 people or where the new infections are increasing at a fast pace*” (SA Coronavirus, n.d.).

Due to the restrictions imposed on individuals during the pandemic, the Health Professions Council of South Africa (HPCSA) had to revise its guidelines on the provision of online health services. In its original General Ethical Guidelines for Good Practice in Telemedicine, the HPCSA discouraged health practitioners from routinely servicing their patients virtually (Health Professions Council of South Africa, 2014). In a recent notice issued on March 26, 2020, in response to COVID-19, the HPCSA pronounced that “telehealth is only permissible in circumstances where there is an already established practitioner-patient relationship, except where telepsychology and/or telepsychiatry is involved; in which case, telehealth is permissible even without an established practitioner-patient relationship” (Health Professions Council of South Africa, 2020). This new development provides an opportunity for psychologists to explore new avenues for providing mental health services for their clients now and, possibly, in the future. As a result of this reform, some telehealth platforms are now inviting health professionals to attend webinars that offer some form of training on how to provide telehealth services, including teletherapy and counseling (for example, see www.ezmed.solutions and www.cgm.com/za). The medical insurance companies have also come out in support of telehealth services by assuring psychologists and other health professionals that they will reimburse the service providers for this alternative service [for e.g., see Discovery Health (n.d.) and GEMS (n.d.)].

### Possible Actions in Stage 2

#### Brazil

There is a COVID-19 Clinical Care Protocol for Primary Care, which does not provide psychological care for these patients (Brazilian Ministry of Health, 2020a), despite the number of psychologists in SUS. Currently, psychologists working in primary care (UBSs) interrupted their routine care (e.g., guidance groups) and performed only welcoming, referring patients who required mental health care to the Psychosocial Care Centers (CAPS) (Brazilian Ministry of Health, 2020b).

CAPS also changed their routine during the pandemic. There is a dispensation of psychiatric medication with a longer term (e.g., for 3 or 6 months, depending on the characteristics of the patients), constant monitoring (weekly or fortnightly) by the technicians (e.g., psychologists), weekly individual attendance for severe cases only (with personal protective equipment) (only one patient and one companion at a time) or home care, after screening for COVID-19 symptoms.

Health institutions (e.g., hospitals) started to provide psychological care for families of inpatients or those who died as a result of COVID-19 and health professionals who care for these patients. Networks were organized to provide psychological assistance for health professionals by universities, associations, and groups of psychologists. Besides, starting in May 2020, online psychological care by SUS is foreseen for health professionals (FIOCRUZ, 2020), which is already happening in some teaching hospitals in the country.

It is expected that, in the second phase of the pandemic, Brazilian Society for Psychology and other scientific societies will continue to support the work of psychologists facing the new challenges. The aim is to improve professional skills and competencies, providing training and supporting the development of technologies and service protocols. Moreover, the scientific production in psychology in the context of COVID-19 must reach the professional psychologists and benefit the population. Communication about the professionals' practice and increased accessibility to psychological services through psychoeducation and telepsychology should be sought.

#### Russia

Representatives of RPS, as well as researchers all over the world, are actively engaged in monitoring the mental conditions of various population groups across Russia, identifying factors that are crucial to the successful accommodation to the period of self-isolation and building strategies for future. RPS, in collaboration with the leading Russian universities supported by The Ministry of Science and Higher Education of the Russian Federation, has started a project “Research at Home.” The purpose of this research is to assess psychological well-being among the student population (*N* = 6,550) to develop evidence-based recommendations to decrease negative effects and increase positive consequences of self-isolation. The results obtained showed no differences in the levels of psychological well-being, significantly lowered levels of depression, anxiety, and stress during the period and higher levels of anxiety and stress during the period. It was shown that studying the impact of the pandemic on the mental health of students requires attention to possible dynamics of their mental state within relatively short periods and gender differences.

#### India

In this stage of the pandemic, the focus is more on dealing with the pressures of the situation, which are mostly socio-structural. In the coming months, the focus has to shift to the psychological needs of people. The emotional impacts of what migrant workers are going through may be a major issue to deal with. The people are walking back to their villages in groups. The social, emotional, and economic costs are far more serious in developing countries like India as compared with developed nations. In the next few months, these vulnerable sections of the society will need more psychosocial support. The government has to seriously look at the role of psychologists in providing the psychosocial and emotional support. The psychology associations have to come forward in providing more mental health-related services and training of psychologists to improve situational skills and competences to increase the accessibility of psychological services.

#### China

The COVID-19 pandemic in China has gone through two phases. The first phase is for protecting and saving lives. Medical treatments are more important than psychological service. But, in the second phase, about 2 months later, psychological reaction or even psychological symptoms, or even mental disorders, especially PTSD, have emerged. For example, those who have witnessed the loss of lives, especially for medical doctors and nurses who had been working in arch hospitals. They knew how vulnerable humankind was in facing the virus. So Division of Nursing Psychology and Division of Medical Psychology, of the CPS, have been working very hard to provide services for medical staff, especially in the first 2 months.

In the first month, it is quite normal for people to react either mentally or behaviorally in stress. Then, in the second month, the third month and, now, the fourth month, upon the relief of the lockdown in May, life returned to the normal schedule in China. So it is a good time for us to reflect on our work, reflect on ourselves, to consider what psychologists can be of help in a future situation like this.

The CPS has done a lot since the lockdown because of COVID-19, the beginning of the pandemic. In the past 4 months, the Standing Committee of the CPS have met twice to make the strategic plans and arrangements of psychological support at the national level, during the lockdown, and after it was relived.

Wenchuan earthquake in 2008 provided experience in psychological support in crisis intervention. Mental and behavioral reactions to a crisis like SARS and COVID-19 pandemic are varied but normal. In the second phase, trauma-related symptoms emerged and can become prominent. Therefore, CPS developed a three-level strategy for psychological service in the COVID-19 pandemic. The three-level-responding strategy to the pandemic brought up by the CPS included the following aspects. (1) Firstly, recruit psychologists with competence. CPS developed competence-enhancement programs for clinical psychologists right after the lockdown, in the end of January. The registration system of CPS has kept training registered psychologists, especially for crisis intervention to improve their competence in providing qualified services for all those in need. (2) Secondly, developing a response system in cooperation with governmental agencies. CPS work together with government agencies, NGOs, and foundations. They try to make sure that the strategic plans are active and sustainable. (3) Thirdly, target key groups in need of psychological service. CPS work with hospitals, schools, and community staff to find the key groups who have psychological problems, mental disorders, or even PTSD.

All institutions have voluntarily done their work. Psychologists set up hotlines, sent out teams working on the frontline, especially Wuhan City. And some of them edited books, developed apps and online programs for psychological services and counseling, and online psychotherapy and teletherapy.

Psychological services have been mentioned by China President Xi Jin-ping in his talks three times since the lockdown. Psychologists have been integrated in the work team for crisis intervention at both the national level and the provincial level. Medical teams were recruited in other provinces and sent to Wuhan or other cities in Hubei Provinces, and psychologists were included in those teams. Ten psychologists had been recommended and included in the National Team of Crisis Intervention, leading by the China Ministry of Crisis Intervention.

Psychological support has been provided in many ways. Counseling or psychotherapy has been provided for free all around China, especially for inhabitants in Wuhan City. Both Division of Personality and Division of Counseling or Clinical Psychology of CPS are affiliated to Central China Normal University, which is located in Wuhan City. Many colleagues working in the School of Psychology there have become the key team members working in the epicenter ever since the lockdown. Almost all universities with a department or school of psychology set up hotlines; for e.g., ShanDong Normal University, located in Ji'nan City, the capital of Shandong province, provides hotline service for the general population in Shandong province. A similar case holds for almost all capital cities of provinces across China.

For knowledge dissemination, many books have been edited, and many web pages have been developed. These can provide psychological knowledge and skills, helping people to adjust and cope with the COVID-19 pandemic. Technology was also employed to assist mental health. Different self-help apps have been developed to help general population online. Also, a wearable wristband with real-time physiological data acquisition, was used by nurses and doctors who work in frontline hospitals. The wristband recorded physical health parameters, mental health parameters and physical activity, providing a quick screening assessment of their (mental) health status.

The COVID-19 outbreak happened during the spring festival, which is a very unique opportunity for family members gathering together. And family dynamics have become a very strong basis of coping with the pandemic. The interpersonal relationship has never been so intensively tied. In this context, CPS has launched a Peace Mind Program, initiated by the Division of Crisis Intervention and Division of Psychological Institutions, together with the Institute of Psychology, Chinese Academy of Sciences. The Peace Mind Program has begun on January 28, right after the Spring Festival. They provided 94 lectures for either professional psychologists or for common civilians to disseminate psychological knowledge and skills. Online psychological assistance was also provided for different groups and professions, especially for policemen, medical professionals, and social workers in communities. We provide a hotline where people can find help or counseling for themselves or for their relatives.

Another initiative, so-called “Thousands of Psychological Institutions for the Peaceful Mind,” is a volunteer recruiting program. The program has recruited about 3,000 psychologists, and more than 400 institutions were involved in the program.

Altogether, 454 organizations were recruited in the Peace Mind Program to provide service in different settings. More than 1,300 medical staff received psychological services, including group therapy, an audio program for stress management. Two thousand four hundred sixty-three institutions have been provided psychological counseling services. More than 4,220 counseling sessions have been provided for individuals. Colleagues in China have composed more than 5,330 essays for knowledge dissemination to the public. For hotlines, nearly 100,000 counseling calls have been received; 77,000 online counseling inquiries have been provided. One hundred seventy-five thousand joined the 1-week program through the APP we developed, with 70% of the users finished the whole program. We also developed a self-evaluation questionnaire, and 187,000 individuals finished the online evaluation. For the video and audio program, we published online, there are more than 1 million views for the lectures, and more than two million play for the audio programs.

Research and project funding has been provided for those initiatives. All work we have done is to find the key groups in need. We realized that there is, still, a lot of work to be done. We hope that international communication and cooperation will give us more understanding of the mental and behavioral reaction to the COVID-19 pandemic and to the crisis in general.

For psychologists, we need to study more on mental and behavioral reactions in a crisis like this and how to provide effective services in the future. For psychologists in China, teamwork, especially work with government agencies and NGOs at different levels from national, provincial, city, and community levels, is very important.

Psychology as a profession in China needs legislation. Psychology as a discipline is very well-developed, but the legal status of the professional psychology profession is not quite stable. We have two kinds of mental health services: one is represented by psychiatrists and the other one is represented by clinical psychologists. These two versions of mental health services, of course, differ in many ways.

#### South Africa

As part of the comprehensive national response, the South African government is implementing an eight-stage process to manage the COVID-19 pandemic (see Table 2). The eight stages are: preparation; primary prevention; lockdown; surveillance and active case-finding; hotspots; medical care (for the peak period); bereavement and the aftermath; and ongoing vigilance (SA Coronavirus, n.d.). The first two stages (i.e., preparation and prevention preceded the lockdown (Stage 3), which also coincided with alert Level 5 of the risk-adjusted response. What is to be done during Stage 4 (surveillance and active case-finding) coincides with alert Level 4 of the risk-adjusted response. During this stage, currently in place during the month of May 2020, the focus is on the community, intending to conduct door-to-door screening, testing, isolation, and contact tracing.

**Table 2:** Eight-stage process to manage the COVID-19 pandemic [adapted from SA Coronavirus (n.d.)].

**Stage**	**Stage 1: preparation**	**Stage 2: primary prevention**	**Stage 3: lockdown**	**Stage 4: surveillance and active case-finding**	**Stage 5: hotspots**	**Stage 6: medical care (for the peak period)**	**Stage 7: bereavement and the aftermath**	**Stage 8: ongoing vigilance**
Actions	• Community education • Establishing lab capacity • Surveillance	• Social distancing & hand-washing • Closing schools and reduced gathering • Close the borders to international travel	• Intensifying curtailment of human interaction	• The Community response: door-to-door screening, testing, isolation and contact tracing The Community response: door-to-door screening, testing, isolation and contact tracing	• Surveillance to identify & intervene in hotspots • Spatial monitoring of new cases • Outbreak investigation & intervention teams	• Surveillance on case load & capacity • Managing staff exposures and infections • Building field hospitals for triage • Expand ICU bed and ventilator numbers	• Expanding burial capacity • Regulations on funerals • Managing psychological and social impact	• Monitoring Ab levels • Administer vaccines, if available • Ongoing surveillance for new cases

As from the beginning of June 2020, the country will transition into Stage 5, which coincides with alert Level 3 of the risk-adjusted response. Looking at Table 2, it is evident that psychologists will have a big role to play in Stage 7 (bereavement and the aftermath). With psychologists mobilized to play a role through structures, such as the HWCN, it does appear that the population will be able to receive some form of psychological services into the future as the pandemic continues to escalate.

## Discussion/Conclusion

COVID-19 provided the context for the first comparative analysis of psychology status in the BRICS nations. As directors of national scientific societies, the authors have worked for the past 3 years to develop BRICS Union of Psychologists, before COVID-19. We have also worked on international communication and cooperation ever since the outbreak of COVID-19. For example, we have participated in the zoom meeting hosted by American Psychological Association at least twice a week, which aims at collecting information from colleagues around the globe.

In the comparative analysis of the actions of psychology in the BRICS countries, the first question that stands out is that the political and economic management of each country influenced the modus operandi of each representation of psychology. Psychological societies received support, encouragement, and even a direct political call for public policy actions for crisis management, psychoeducation, first psychological aids, and active listening directed to the public (population, health professionals, and patients). Moreover, all societies have engaged in confronting COVID-19 in public health at various levels, considering the impacts on mental health.

Although psychology acts to promote mental health in BRICS, only in Russia that psychology had a strong role in remote education in these early months of COVID-19. On the other hand, psychology in China was quickly organized, and this will speed up the required legislation and professional preparedness. In China and Russia, the scientific societies worked closer and integrated with the government. In Brazil, psychologists are integrated into the public health system, and a large number of psychologists are registered in a federal regulatory council of their own, but telepsychology is underdeveloped; state governments are working with Federal and State Universities to provide telepsychology during COVID-19. In India and Brazil, there is a prevalent sense of dichotomized world view, in contrast with the strong interpersonal strengthening described in China.

Countries with large social inequalities faced more socioeconomic gaps that affect mental health, further straddling the health system weaknesses, as shown by Brazil, India, and South Africa. The interface between the socioeconomic and political moment might have contributed to the exacerbation of mental health issues in the early months of COVID-19. The various psychology organizations in these countries have tried to organize themselves independently or seeking to sensitize governments to articulate actions in favor of minority groups (indigenous people, people in situations of socioeconomic vulnerability, and people in rural areas). In general, interventions introduced included payment of a social relief grant or of a distress grant to the unemployed and an increase in the social grant amount paid to the indigent. Also, technological and/or socioeconomic inequalities in countries like Brazil, India, and South Africa did not allow online services (health, mental health, and education) to reach everyone (rural, indigenous population, the poorest, and the most vulnerable), in contrast to online education in Russia and hotlines, apps and wearables in China.

Despite this deficient access to online services in some of the BRICS nations, the expanded and extensive insertion of the telepsychology happened in all of them. The guidelines on this modality of online intervention were different for each nation and had to be reformulated due to the situation. In Brazil and South Africa, there were regulations being discussed under pressure, reformulated and adapted to the context of COVID-19.

Psychology has different roles in their societies, and the pandemic exacerbated the strengths and exposed some of the weakness. In a long and continuous tradition of Russian psychology, the government actions have a place for education, mental health, and refined identification, and intervention is possible, for instance, in emerging cyberbullying. China, the first country of the epicenter of the pandemic, has been working in this crisis, using previous experience and an organized psychological intervention in many levels of psychological care contextualized, with the support of integrated and expanded political and economic management that helped in facing the emotional issues. India has a prolific amount of actions and a recent mental care policy that will probably catalyze the experiences during the pandemic into better mental health services. Psychology in South Africa has a strong regulatory link with other health professions; as a result, the strategies were set for all health professions, and psychology benefited from that despite the low contingent of psychologists. In contrast, despite of the high contingent of psychologists in Brazil, telepsychology was not stimulated, and the strong public health system did not offer telepsychology to the general population; universities and scientific societies are leading the actions in mental health during COVID-19 in independent initiatives amid a complex political context. In general, psychology in BRICS has been making pioneer contributions during COVID-19 times.

Psychoeducation of the general population and building competencies of psychologists to work during and after the crisis are the main aims in BRICS. The different approaches of each country are related to the role of psychology in society, equity, and social context. The use of digital platforms and telepsychology has been one important resource that gives visibility to psychology and expands mental health care. In general, it is expected that there will be an increased role of psychologists in providing cultural sensitive intervention and information on socioemotional skills.

## Author Contributions

KA and LB: design, integration of the authors' comments, and final manuscript. KA, LB, MM, MS, AP, MT, JM, AV, PS, BH, and TS: writing the draft. All the authors contributed to manuscript revision, read, and approved the submitted version.

## Conflict of Interest

AP was employed by Bee Touch. The remaining authors declare that the research was conducted in the absence of any commercial or financial relationships that could be construed as a potential conflict of interest.

## Footnotes

^1^Modesto, J. G., Keller, V. N., Rodrigues, C. M. L., and Lopes, J. L. S. (2020). The moderating role of belief in a just world on the association between belief in fake news and prevention behaviors during the Covid-19 pandemic (Manuscript submitted).
